# Right Atrial and Bicaval Reconstruction for Cardiac Sarcoma

**DOI:** 10.1016/j.jaccas.2024.102978

**Published:** 2024-12-18

**Authors:** Nitish K. Dhingra, Abdullah H. Ghunaim, Dambuza Nyamande, Abdulaziz M. Alhothali, Robert J. Cusimano

**Affiliations:** Division of Cardiac Surgery, Toronto General Hospital, University of Toronto, Toronto, Ontario, Canada

**Keywords:** cardiac tumor, percutaneous biopsy, sarcoma, surgical resection

## Abstract

Sarcomas represent the most common primary cardiac malignancy. A poor prognosis can be improved with multimodal management including aggressive surgical reconstruction in combination with neoadjuvant or adjuvant therapy. We present the case of a primary cardiac sarcoma to describe our approach to a more radical right atrial and bicaval reconstruction.

## Graphical Abstract

**Figure:**
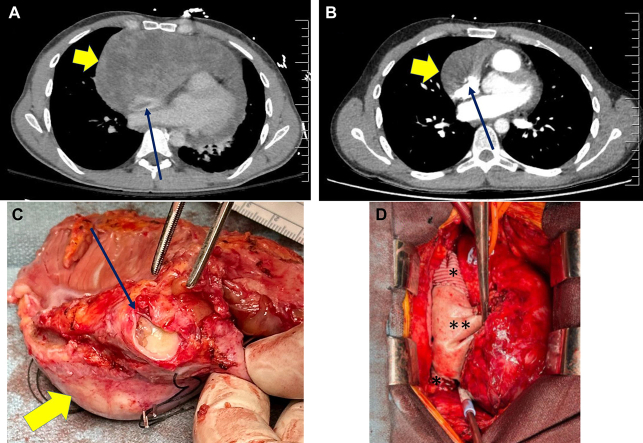

A 27-year-old patient presented with chest pain and dyspnea. A chest x-ray showed a significantly enlarged cardiac silhouette which led to a noncontrast chest computed tomography (CT) scan that suggested pericardial effusion. Subsequently, a pericardiocentesis was performed (bloody fluid, negative for malignancy) with resolution of symptoms. Four weeks later, his symptoms recurred. This time, after drainage (again bloody), he had a contrast CT, which showed a large lobulated mass of the mediastinum and right atrium (RA) suspicious for a sarcoma (Figure 1A). Echocardiography confirmed the above findings. A CT-guided percutaneous biopsy confirmed synovial sarcoma and, after a full course of chemotherapy, repeat imaging showed a resectable tumor (Figure 1B). Thus, we proceeded with resection.

**Take-Home Message**

• The addition of the venae cavae to right atrial resections to improve the chances of a complete (R-0) resection is possible and should be considered in malignant lesions in the region.

**Figure 1:**
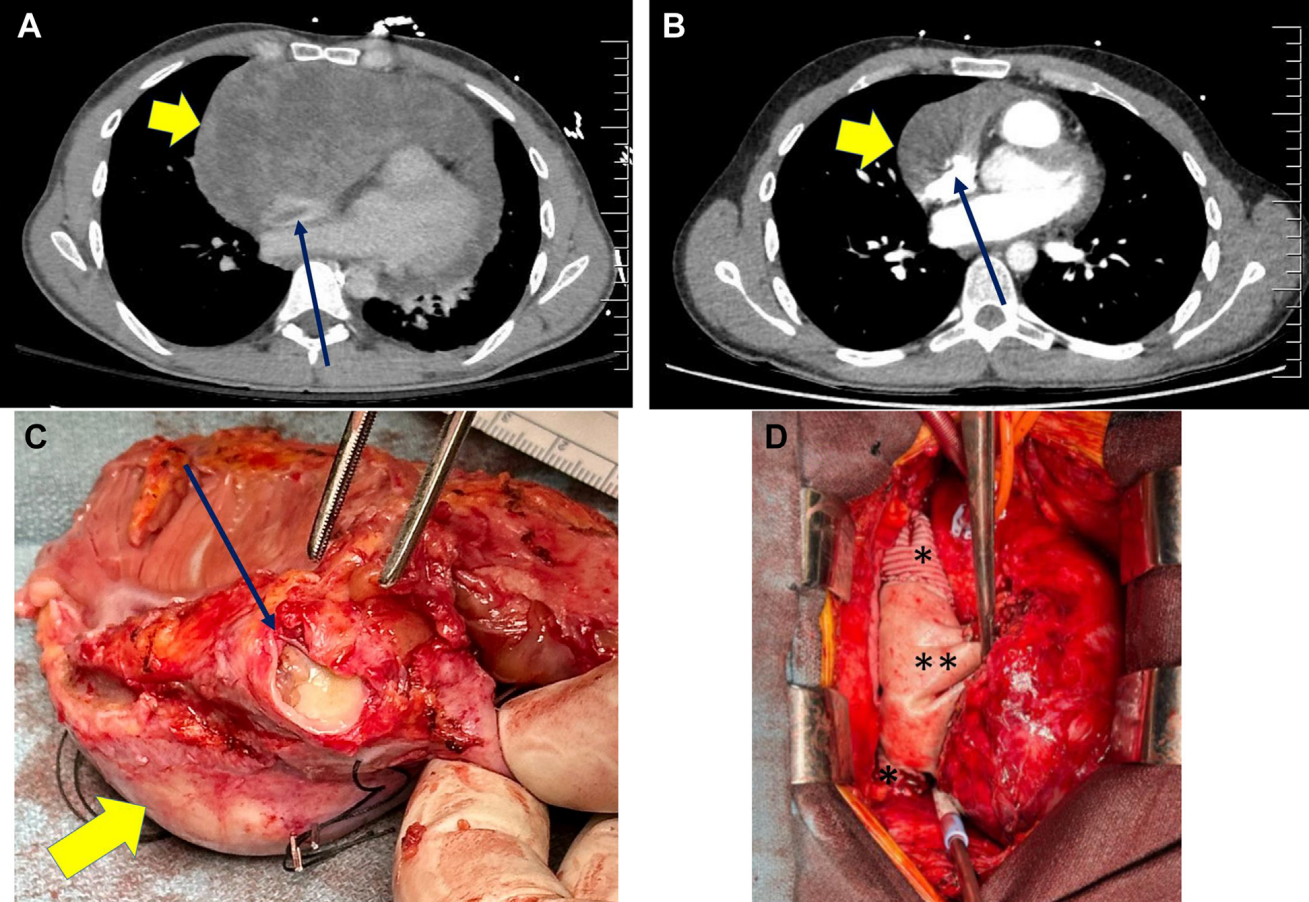
The Tumor After Drainage and After Chemotherapy, the Surgical Specimen, and the Intraoperative Image (A) The tumor after drainage of the pericardial effusion. The short arrow shows the main tumor and the long arrow shows the superior vena cava (SVC) extension of the tumor in A, B, and C. (B) After neoadjuvant chemotherapy (immediately preoperative). (C) The surgical specimen looking through the SVC from a superior view. The SVC was resected in its entirety. (D) Intraoperative image of bicaval and right atrial reconstruction following cardiac sarcoma resection. The neo–right atrium was constructed from bovine pericardium (∗∗), and the Dacron graft can be seen at the SVC and inferior vena cava diaphragm junction (∗).

The patient was taken to the operating room and a median sternotomy performed. We found a large tumor involving the pericardium adjacent to the RA. It extended into the superior vena cava (SVC) and inferior vena cava (IVC) and onto the anterior right ventricle. There was a significant amount of edema which helped resection of the mass in some locations. Using a high SVC with femoral venous cannulation along with ascending aortic cannulation, we resected the right atrium, pericardium, SVC, beveled at the azygous vein and IVC to the pericardium, en bloc (Figure 1C). The tricuspid annulus was spared by the tumor (frozen section confirmed), was structurally normal and competent; therefore, it was left alone. After the radical resection, a 28-mm Dacron graft was firstly used to reconstruct the SVC to the pericardial reflection. Following this, a window was made into the medioanterior aspect of the graft and a patulous bovine pericardial patch was used to reattach the heart to the graft, suturing to the annulus and coronary sinus proper (origin resected) (Figure 1B). Intraoperative transesophageal echocardiogram demonstrated no new valvular or ventricular dysfunction. Tumor resection margins were negative on final histopathologic examination. Given that we resected the sinoatrial node, an epicardial dual chamber pacer was placed. The patient tolerated the procedure well and was discharged without complication.

Primary cardiac malignancies are associated with very poor prognosis, with only 10% of patients surviving past 24 months with medical therapy alone.^1^ Transplant offers no advantage in primary sarcoma.^2^ Aggressive surgical resection and reconstruction in combination with adjuvant or neoadjuvant (as in this case) therapy offer the best opportunity for negative margins and outcomes.^3^ Although reconstruction of the right atrium with or without the tricuspid valve can be accomplished, removing the SVC and IVC with the specimen increases the likelihood of an R-0 (complete microscopic) resection. The use of a Dacron graft made great vessel resection possible. Any material that the team is familiar with can be used. The obligatory loss of RA function can be minimized by providing a patulous RA, although this has not been tested.

As demonstrated in this case, fluid obtained from pericardiocentesis yields negative cytology in the majority of malignancy-related effusions and can thereby provide false reassurance.^4^ As such, we recommend routine use of contrast-enhanced chest CT scans following pericardiocentesis for all patients found to have bloody pericardial effusion, regardless of cytology. Prompt diagnosis remains a crucial predictor of long-term survival.

## Funding Support and Author Disclosures

The authors have reported that they have no relationships relevant to the contents of this paper to disclose.
